# Redirecting T Cells to Ewing's Sarcoma Family of Tumors by a Chimeric NKG2D Receptor Expressed by Lentiviral Transduction or mRNA Transfection

**DOI:** 10.1371/journal.pone.0031210

**Published:** 2012-02-15

**Authors:** Manfred Lehner, Gabriel Götz, Julia Proff, Niels Schaft, Jan Dörrie, Florian Full, Armin Ensser, Yves A. Muller, Adelheid Cerwenka, Hinrich Abken, Ornella Parolini, Peter F. Ambros, Heinrich Kovar, Wolfgang Holter

**Affiliations:** 1 Laboratory of Cellular Therapy, Children's University Hospital, Universitätsklinikum Erlangen, Friedrich-Alexander-Universität Erlangen-Nürnberg, Erlangen, Germany; 2 Department of Dermatology, Universitätsklinikum Erlangen, Friedrich-Alexander-Universität Erlangen-Nürnberg, Erlangen, Germany; 3 Institute for Clinical and Molecular Virology, Friedrich-Alexander-Universität Erlangen-Nürnberg, Erlangen, Germany; 4 Lehrstuhl für Biotechnik, Department of Biology, Friedrich-Alexander-Universität Erlangen-Nürnberg, Erlangen, Germany; 5 German Cancer Research Center, Heidelberg, Germany; 6 Center for Molecular Medicine Cologne (CMMC), University of Cologne, Cologne, Germany; 7 Centro di Ricerca E. Menni, Fondazione Poliambulanza Istituto Ospedaliero, Brescia, Italy; 8 Children's Cancer Research Institute (CCRI), St. Anna Hospital, Vienna, Austria; University of London, St George's, United Kingdom

## Abstract

We explored the possibility to target Ewing's sarcoma family of tumors (ESFT) by redirecting T cells. To this aim, we considered NKG2D-ligands (NKG2D-Ls) as possible target antigens. Detailed analysis of the expression of MICA, MICB, ULBP-1, -2, and -3 in fourteen ESFT cell lines revealed consistent expression of at least one NKG2D-L. Thus, for redirecting T cells, we fused a CD3ζ/CD28-derived signaling domain to the ectodomain of NKG2D, however, opposite transmembrane orientation of this signaling domain and NKG2D required inverse orientation fusion of either of them. We hypothesized that the particularly located C-terminus of the NKG2D ectodomain should allow reengineering of the membrane anchoring from a native N-terminal to an artificial C-terminal linkage. Indeed, the resulting chimeric NKG2D receptor (chNKG2D) was functional and efficiently mediated ESFT cell death triggered by activated T cells. Notably, ESFT cells with even low NKG2D-L expression were killed by CD8^pos^ and also CD4^pos^ cells. Both, mRNA transfection and lentiviral transduction resulted in high level surface expression of chNKG2D. However, upon target-cell recognition receptor surface levels were maintained by tranfected RNA only during the first couple of hours after transfection. Later, target-cell contact resulted in strong and irreversible receptor down-modulation, whereas lentivirally mediated expression of chNKG2D remained constant under these conditions. Together, our study defines NKG2D-Ls as targets for a CAR-mediated T cell based immunotherapy of ESFT. A comparison of two different methods of gene transfer reveals strong differences in the susceptibility to ligand-induced receptor down-modulation with possible implications for the applicability of RNA transfection.

## Introduction

A subgroup of patients with Ewing's sarcoma family of tumors (ESFT) is still threatened by a poor long term prognosis. Despite modern multimodal therapy (chemotherapy, radiation, and surgery) ESFT relapse in about 30% of patients with localized disease. Long-term survival of those who relapsed and of patients with metastatic disease at diagnosis is currently below 30% [1]–[3]. Therefore, new treatment options are needed.

Tumors cells frequently up-regulate “stress” induced ligands recognized by the NK cell activating receptors DNAM-1 (CD226) and NKG2D (CD314), whose ligands have been found recently also on ESFT cells [4]. Therefore, the infusion of NK cells has emerged as a promising new treatment strategy for malignant tumors in general and has been also suggested for the treatment of ESFT [4], [5]. NK cells due to their innate specificity allow tumor targeting without extensive *ex vivo* modification, and have not been reported to cause auto- or allo-immune side-effects following transfusion even across MHC barriers [6], [7]. CD8^pos^ T cells, on the other hand, are characterized by the capacity to differentiate into effector cells or long term memory cells, and have been adopted in the past with a broad range of new antigenic specificities by receptor transfer (for review see [8]).

NKG2D recognizes several ligands (MICA, MICB, ULBP-1 to ULBP-6) with only restricted expression in normal tissues [9], [10]. Utilizing this receptor for redirecting T cells, Sentman and co-workers recently reported the construction of an NKG2D-based chimeric T cell antigen receptor (CAR) and demonstrated its efficacy against a variety of malignant cells *in vivo* and *in vitro* [11]–[14]. We wanted to further develop this approach for targeting ESFT and decided to construct a similar NKG2D-based CAR, but - based on the known 3D-structure of NKG2D - focused on reengineering the extra-cellular domain of NKG2D to allow its fusion in type I membrane protein orientation to the signaling domain of a second generation CAR, which contains also CD28 for enhanced signal transduction [8], [15], [16]. Here we report that this new chNKG2D successfully allowed us to establish a T cell based approach for efficient ESFT cell elimination.

For expression of this chimeric receptor we compared two different methods of gene transfer, namely mRNA transfection and lentiviral transduction, since both methods are currently considered for clinical application. Lentiviral mediated gene transfer mediates stable transgene expression and therefore is attractive, because the clinical effectiveness of adoptive tumor therapy has been correlated with the persistence of transferred cells [17]. On the other hand, the transient nature of receptor expression following mRNA transfection might be an advantage under the aspects of safety and other regulatory issues. Although mRNA transfection thus has important benefits, we asked how such transient expression influences receptor stability following target cell recognition.

## Materials and Methods

### Ethics Statement

Written informed consent was obtained from all healthy voluntary donors involved in the study (commissioned by the local ethic committee of the Friedrich-Alexander Universität Erlangen-Nürnberg, Ethics proposal 2247, 10.07.2000). All data were analyzed anonymously.

### Cells

Primary human mononuclear cells (MNCs) were obtained from blood of healthy voluntary donors (commissioned by the local ethic committees) by density gradient centrifugation using endotoxin-free Ficoll-Paque-PLUS® (Amersham Pharmacia Biotech AB, Uppsala, Sweden). CD8^pos^ and CD4^pos^ T cells were isolated from MNCs by negative magnetic selection using microbeads (Miltenyi Biotec, Bergisch-Gladbach, Germany). T cells were cultured in R10-IL2 medium consisting of RPMI 1640 supplemented with 10% FBS and 100 U/ml IL-2 (Millipore, Billerica, MA). 293T cells and the myeloid cell lines MONO-MAC-6, U-937, HL-60, NB-4, OCI-AML5, MV4-11, KG-1a, and THP-1 were obtained from the German Resource Center for Biologic Material (DSMZ, Braunschweig, Germany), the glioblastoma cell line U-373 was obtained from ATCC. The ESFT cell lines STA-ET-1, -2, -3, -6, -8.2, -10, -11, ER-ESFT-1 were generated in house [18], [19], CADO-ES1, TC-71, WE-68, VH-64 were provided by Frans van Valen [20]. All ESFT cell lines were maintained in culture dishes, which were pre-coated for 2 hours at RT with a 0.1% gelatin solution in PBS. Murine cell lines stably, expressing either human ULBP2 or MICA, were generated by transduction of Ba/F3 pre-B cells with a pMX-pie based retroviral expression vector [21]. All cell lines were maintained in RPMI 1640 supplemented with 10% FBS and IFN-γ when indicated.

### Generation of chNKG2D

The extracellular portion of human NKG2D (aa82–216) was amplified by PCR from a plasmid containing the NKG2D cDNA sequence by using the primers 5′-cgaatagt*ccatggaagctt*a**gccacc** atgccgctgctgctactgctgcccctgctgtgggcaggggccctggctatgggtacctctagattattcaaccaagaagttcaaattccc-3′ containing a Kozak-sequence (bold), CD33 signal peptide sequence (underlined), restriction sites (italic) of *Nco*I and *Hind*III downstream; 5′-gatgtcac**ggatcc** ccgccaccgcccgatccaccacctcctgaca-cagtcctttgcatgcagatg-3′ with a *Bam*HI-site (bold) and the Gly4Ser-Gly4Asp-linker (underlined). The PCR product was cloned by *Nco*I×*Bam*HI restriction into the retroviral vector pBullet 607 containing the IgG1-Fc/CD28/CD3ζ backbone [22]. For *in vitro* transcription, the NKG2D portion was recloned from the pB607/NKG2D vector into the pGEM4Z-CEA vector, also containing the IgG1-Fc/CD28/CD3ζ backbone, by *Nco*I×*Bam*HI restriction. The NKG2D portion was additionally recloned from the pB607/NKG2D vector into a derivate of the vector pST1 (provided by U. Sahin) for transcription of “RNA #2”. The scFv-portion of a Cytomegalovirus glycoprotein gB (CMV-gB)-specific IgG1-Fc/CD28/CD3ζ-CAR-construct [23] was replaced by *Hind*III×*Bam*HI restriction. After recloning, the polyA tail was found to be shortened from 120 to approximately 100 A nucleotides as estimated by *Eco*53KI restriction. This vector was linearized for *in vitro* transcription with LguI to produce a polyA-tail devoid of non-A nucleotides. For lentiviral expression NKG2D/IgG1-Fc/CD28/CD3ζ was excised from pB607/NKG2D by *Hind*III×*Bsp*EI restriction and cloned into the *Pme*I site of the lentiviral plasmid pWPI (D. Trono, Geneva).

### *In vitro* transcription and RNA-electroporation

*In vitro* transcription and electroporation was performed as previously described [24]. Briefly, *in vitro* transcription was performed with linearized pGEM4Z-NKG2D or pST1-NKG2D using the mMESSAGE-mMACHINE-T7 Ultra kit (Applied Biosystems/Ambion) followed by polyadenylation. The vector pST1 was developed by Holtkamp et al. [25] to allow *in vitro* transcription of a more stable mRNA and the kit used for *in vitro* transcription was optimized for a more efficient translation initiation by using the modified anti-reverse cap analog (ARCA, 7-methyl(3′-O-methyl)GpppG)m7G(5′)ppp(5′)G). RNA from a cognate CMV-gH-specific IgG1-Fc/CD28/CD3ζ-CAR (Goetz G., unpublished) served as control. Electroporation was performed with 10 µg RNA/100 µl Opti-MEM containing ≤6×10^6^ CD8^pos^ or CD4^pos^ T cells either immediately after isolation or 12 days after activation with an anti-CD3-antibody (clone OKT3). Anti-CD3-activation was performed by plating 0.2×10^6^ T cells/ml on wells pre-coated with 10 µg/ml of anti-CD3-antibody in R10-IL2 medium. After two days the cells were transferred to fresh wells without anti-CD3-antibody. Half of the medium was replaced twice a week. Electroporated T cells were further cultured in R10-IL2 medium and used for functional analysis one day after RNA transfection.

### Production of lentivirus and transduction of T cells

293T cells were seeded in 75 cm^2^ flasks and transfected using Lipofectamine 2000 (Invitrogen Corporation, Bethesda, MD) according to manufacturer's instructions. The pWPI vector plasmid containing the chNKG2D was cotransfected together with psPAX2 (packaging) and pMD2.G (VSV-G, envelope) in a ratio of 4∶3∶1 (total 15 µg plasmid) with 12.5 µl Lipofectamine 2000 per flask. The supernatants were collected 48 hours after transfection by centrifugation (10 min, 2000 rpm) to remove cell debris, and were stored at −80°C until further usage. Prior to transduction primary human cells were activated over night by 2.4 µg/ml PHA in R10-IL2 medium. Transduction was performed by spinoculation of 0.5–1×10^6^ cells with 0.5 ml of virus containing supernatant (minimum 0.5 µg p24; quantified by ELISA), supplemented with 5 µg/ml Polybrene (Sigma), at 1500×g for 4 hours at 33°C. After an additional incubation over night at 37°C the cells were washed twice and further maintained in R10-IL2 medium. For enrichment of chNKG2D^pos^ cells irradiated ULBP2 expressing murine Ba/F3 cells were added 1∶1 in weekly intervals. Control T cells transduced with a pWPI vector containing a cognate CMV-gB-specific CAR [23] were enriched by co-culture with irradiated murine IIA1.6 cells stably expressing CD64 (provided by Jan van de Winkel; University Medical Center Utrecht, The Netherlands). The immunophenotype of transduced T cells was controlled by flow cytometry before functional analysis (data not shown). Only pure CD3^pos^ TCRα/β^pos^ cultures not containing NK cells were used in functional assays seven to ten days after the last stimulation with ligand^pos^ target cells.

### Flow cytometry

Human cells were analyzed by anti-CD3 (clone S4.1), anti-CD4 (clone SK3), anti-CD8 (clone SK1), anti-CD56 (clone NCSM16.2), anti-pan TCR-alpha/beta (clone BMA031), anti-MHC-class-I (clone Tü149), and appropriately labeled IgG1 and IgG2b isotype controls. Expression of chNKG2D was detected either by anti-CD314 (clone 1D11) or via its human IgG1-Fc portion using biotinylated anti-human-Fc (clone JDC-10) and streptavidin-PE. NKG2D-Ls were analyzed with anti-MICA (clone AMO1), anti-MICB (clone BMO2), anti-ULBP1 (clone AUMO2), anti-ULBP2 (clone BUMO1), anti-ULBP3 (clone CUMO3) (all from Axxora Life Sciences Inc., San Diego, CA) and an APC-labeled goat-anti-mouse-IgG used as secondary antibody.

### ELISA

50 000 activated T cells were co-cultured with 100 000 target cells in 0.5 ml of R10-IL2 medium supplemented with 0.3% Sandoglobulin® (CSL Behring, King of Prussia, PA). Co-culture with ESFT cells was performed in gelatin coated wells. Supernatants were harvested after 20 hours and TNF, FasL, and TRAIL were determined by using ELISA kits from Mabtech, Diaclone, and BD Biosciences, respectively, according to the manufacturer's instructions. The figures show total cytokine levels without background subtraction.

### Cytotoxicity assay

The cytoxic activity was analyzed either by determining target cell lysis using a non-radioactive Europium (Eu)-based assay or, if Eu-labeling of target cells was inefficient, by determining apoptosis induction in target cells by flow cytometry. Europium assay: ESFT cells were harvested by trypsin/EDTA treatment and labeled with Eu as previously described [26]. Eu released from 1000 labeled target cells was determined in the supernatants after 4 hours of co-culture. Specific lysis was calculated by the formula: % lysis = (experimental counts minus spontaneous release)×100/(maximum release minus spontaneous release). Annexin V apoptosis assay: Target cells were harvested, washed in PBS and labeled by incubation with 2 µM SNARF-1 carboxylic acid, acetate succimidyl ester (Invitrogen) in PBS for 15 minutes at 37°C. Labeled cells (5000) were preincubated with 1% Sandoglobulin® for 20 minutes and then co-cultured with effector T cells at the indicated ratios in 200 µl R10-IL2 medium in a 96 well round bottom plate. After 5 hours, the cells were harvested by trypsin/EDTA and then supplemented with 1% FBS, 2 mM CaCl_2_, and Annexin V-FITC. The fraction of Annexin V^pos^ cells of SNARF-labeled target cells was determined by flow cytometry (measured in FL4). The percentage of spontaneous Annexin V^pos^ target cells (in absence of T cells) was subtracted as unspecific background and the fraction of “specific” Annexin V^pos^ targets actually induced by T cells was calculated by the formula: % Annexin V^pos^ = (Annxin V^pos^ of co-culture minus spontaneous Annexin V^pos^)×100/(100 minus spontaneous Annexin V^pos^). The fraction of spontaneous Annexin V^pos^ and maximum possible Annexin V^pos^ (100%) cells were corrected by the fraction of actually ligand expressing cells of the stably transfected cell line Ba/F3 (70–90%).

### Statistical analysis

Statistical significance was estimated with the paired two-tailed Student's t test (*** = *P*<0.001, ** = *P*<0.01, * = *P*<0.05, ns = *P*>0.05).

## Results

### Expression of NKG2D-ligands in ESFT

**Figure 1:**
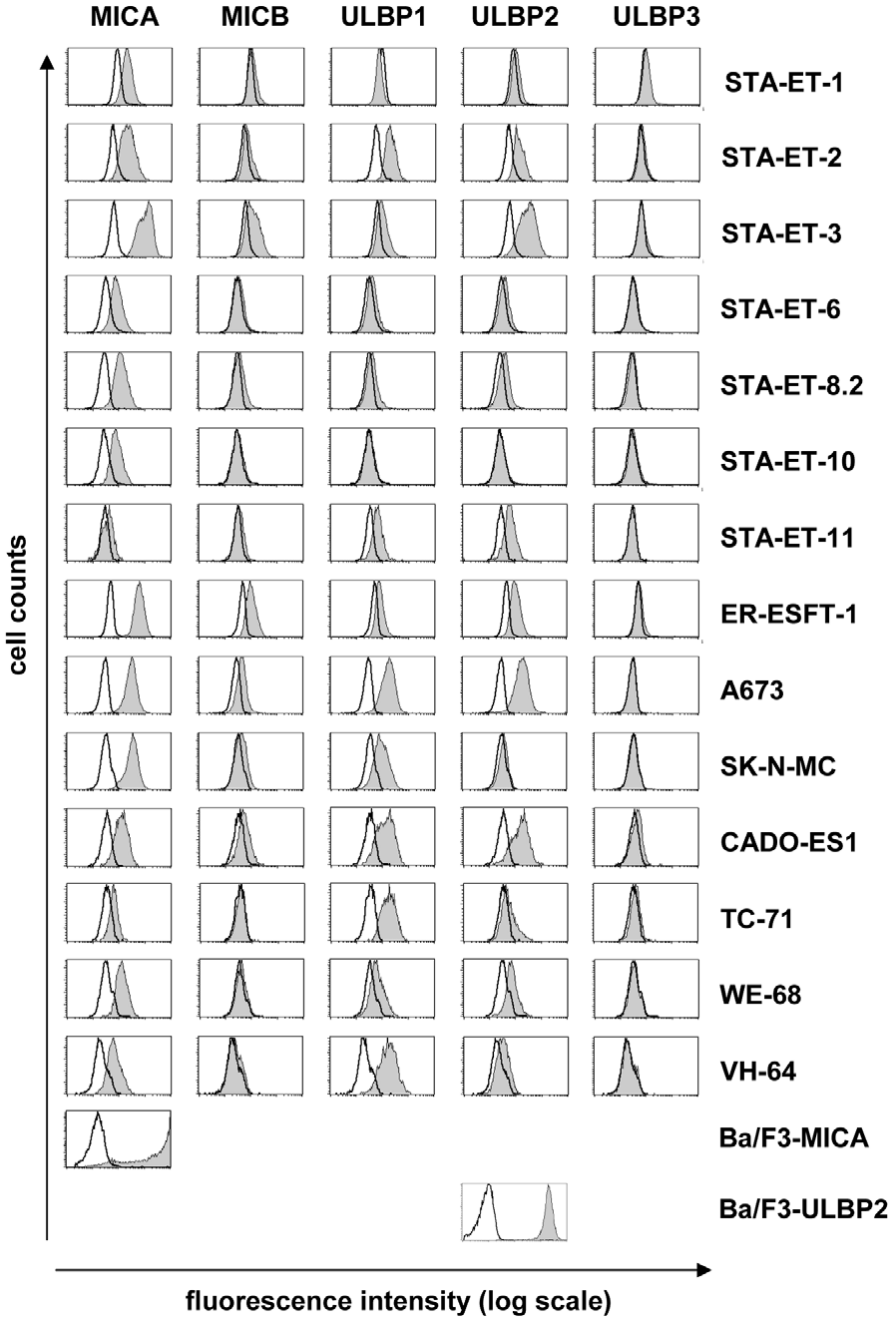
Expression of NKG2D-Ls in ESFT. The histograms show the surface expression of the respective NKG2D-Ls recognized by specific monoclonal antibodies and analyzed by flow cytometry (filled histograms = specific antibodies; open histograms = isotype controls). The two stably transfected murine B cell lines (Ba/F3) expressing either human MICA or ULBP2 are displayed for comparison.

The expression of different NKG2D-Ls has been reported previously for several ESFT cell lines [4]. Here, we screened 14 ESFT cell lines for the expression of MICA, MICB, and ULBP1, ULBP2, and ULBP3 by flow cytometry. The results emphasize that NKG2D-Ls appear to be broadly expressed by ESFT cell lines, although some (STA-ET-1, -6, -8.2, -10, -11) displayed only fairly low ligand expression (Fig. 1). Most cell lines expressed MICA and frequently also ULBP1 and/or ULBP2. MICB was only weakly expressed in some cell lines and ULBP3 was consistently absent. From the data we concluded that NKG2D-Ls might allow targeting of a broad range of ESFT cells.

### Construction of the chNKG2D

**Figure 2:**
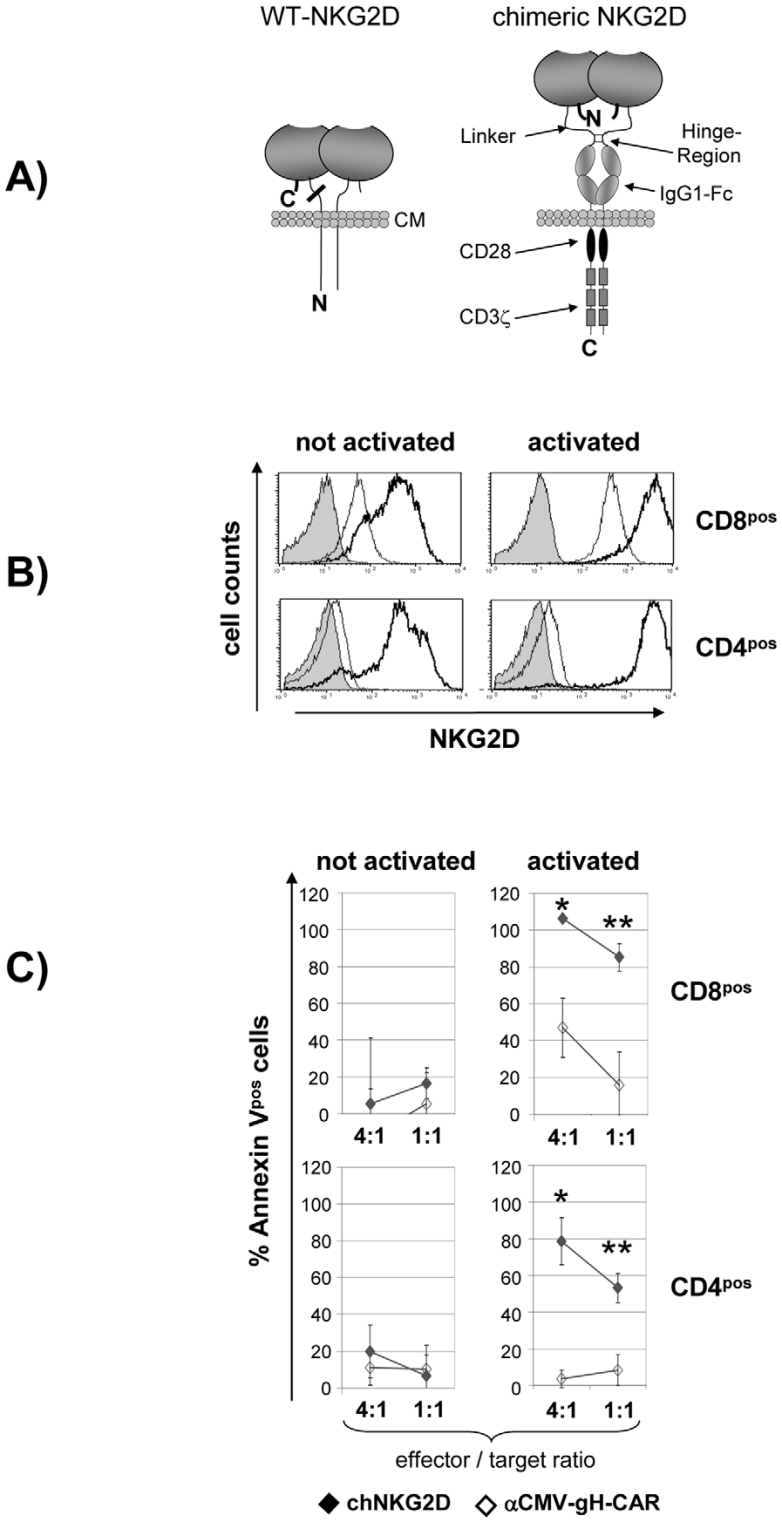
Construction and characterization of the chNKG2D. *A,* The figure schematically illustrates the domain architecture of wildtype NKG2D and chNKG2D (N = N-terminus, C = C-terminus, CM = cytoplasma membrane). The N-terminal truncation of the extracellular NKG2D domain at aa81 is indicated by a bold bar. The native C-terminal end of NKG2D was fused to the N-terminus of the IgG1-Fc/CD28/CD3ζ transmembrane signaling platform creating a transmembrane receptor of type I orientation in which the relative orientation and positioning of the NKG2D ectodomain has not been changed. *B,* efficient transfection and strong surface expression of chNKG2D by electroporation of mRNA into primary T cells. The histograms show the surface expression of chNKG2D 24 hours after electroporation of quiescent or anti-CD3-activated human T cells (thick line, open histogram). Analysis was performed by flow cytometry using an anti-CD314 antibody. The expression of endogenous wildtype NKG2D in T transfected with a CMV-gH-specific CAR is shown for comparison (thin line, open histograms; filled histograms = isotype controls). *C,* chNKG2D triggers cytotoxicity of activated T cells. Quiescent or anti-CD3-activated CD8^pos^ or CD4^pos^ T cells were transfected with RNA encoding either the chNKG2D or a CMV-gH-specific control receptor. One day after transfection, the T cells were co-cultured with dye-labeled murine B cells (Ba/F3-MICA) stably expressing the human NKG2D-L MICA. The diagram shows the percentage of Annexin V^pos^ murine target cells after 5 hours of co-culture at the indicated E∶T ratios (data pooled from three independent experiments using different cell donors; mean ± S.D.). Statistical significance was calculated for the effects of activated T cells expressing the chNKG2D versus T cells expressing the control receptor.

Existing signaling platforms of CARs are derived from type I transmembrane proteins with extra-cellular N- and intra-cellular C-terminus. NKG2D, contrary to most transmembrane proteins, however, is a type II receptor with inverse orientation. Fusion of NKG2D with the existing signaling platforms therefore could alter their configuration and function. To avoid this and to allow future improvements of signaling we tested the option of constructing a chNKG2D with type I orientation. The ectodomain of wildtype NKG2D forms non-covalent homo-dimers and is linked to the transmembrane domain via a 21 amino acid residues (aa) long highly flexible anchor (aa75–95). Analyzing the published crystal structure (Protein Data Bank; accession code 1HYR) revealed that the C-terminal end of NKG2D protrudes from the globular ectodomain in close proximity to the entry site of the membrane anchor (Fig. 2A) [16]. Thus, we hypothesized that the NKG2D ectodomain could be alternatively anchored via a C-terminal linkage without disturbing its relative positioning. This should allow its integration into a CAR composed of a well established IgG1-Fc/CD28/CD3ζ signaling platform with type I orientation [22]. We chose to truncate the NKG2D ectodomain within its native membrane anchor at aa81 thereby removing the transmembrane and intracellular portion of NKG2D and to fuse its C-terminal end (aa216) via an artificial flexible Gly/Ser-linker to the N-terminus of the IgG1-Fc/CD28/CD3ζ molecule. The flexible upper hinge residues (DKTHT) followed by the hinge core residues (CPPC) containing the inter-chain disulfide bonds of IgG1-Fc served as N-terminal acceptor site of the signaling platform [27]. We calculated that a linker consisting of two Gly/Ser-repeats (GGGGSGGGGD; last serine replaced by hydrophilic aspartic acid) would by appropriate to allow sufficient flexibility for homo-dimeric pairing of the extra-cellular NGK2D portions but not to provoke sensitivity to proteolytic degradation. Wildtype NKG2D after translation into the endoplasmatic reticulum is directed to the cell surface via an uncleaved type II transmembrane signal anchor. This anchor was replaced in the chimeric receptor by the type I transmembrane signal anchor of CD28. Furthermore, as type I membrane proteins require also cleavable N-terminal signals for appropriate insertion into the cell membrane, we introduced the cleavable N-terminal signal peptide of CD33, which is removed in the mature protein. Figure 2A displays a scheme of the final mature fusion protein.

### Transfer of chNKG2D into different T cell populations by electroporation of mRNA

T-cell activation might be required for triggering effector functions also in the CD45RO^pos^ T cell subpopulation [28], however, induced proliferation might accelerate the loss of chNKG2D expression due to dilution of transfected mRNA. Thus, we compared resting and activated T cells. CD8^pos^ and CD4^pos^ T cells were purified from peripheral blood and electroporated either directly or 12 days after activation via CD3, when proliferation had declined again. Figure 2B shows the expression of chNKG2D one day after transfection. Detection was done by an anti-NKG2D(CD314)-antibody, which also detects endogenous NKG2D expressed in CD8^pos^ T cells. T cells transfected with a CMV-gH-specific control CAR [23] were thus included in Figure 2B for comparison. ChNKG2D-mRNA-transfected T cells displayed strongly increased anti-NKG2D-staining compared to CMV-gH-CAR controls. Based on peak channel numbers from three independent experiments using cells from different donors, we calculated a 5–9-fold increased chNKG2D expression density in chNKG2D-RNA-transfected activated CD8^pos^ T cells compared to endogenous NKG2D (Fig. 2B and data not shown). The expression of chNKG2D was homogeneous and augmented following cell activation both in CD4^pos^ and CD8^pos^ T cells compared to the resting cells.

NKG2D-Ls are monomeric and are recognized only by the homo-dimerized native NKG2D receptor by asymmetric binding of the α1- and the α2-epitopes of the ligands [16]. Here, we evaluated the functional activity of the novel chNGK2D type. Figure 2C shows that chNKG2D^pos^ T cells can induce apoptosis in a murine cell line stably expressing the NKG2D-ligand MICA (Fig. 1). This experiment also illustrates that resting chNKG2D^pos^ T cells were clearly inferior to CD3-activated chNKG2D^pos^ T cells, which killed their target cells even at an effector∶target (E∶T) ratio of only 1∶1. Importantly, apoptosis in MICA^pos^ targets was induced both by chNKG2D-expressing CD8^pos^ as well as CD4^pos^ T cells. The data so far confirmed that the novel recombinant chNKG2D is functional and that RNA transfection allows high level expression on the surface of primary T cells.

### ESFT cells are killed by T cells transfected with chNKG2D mRNA

**Figure 3:**
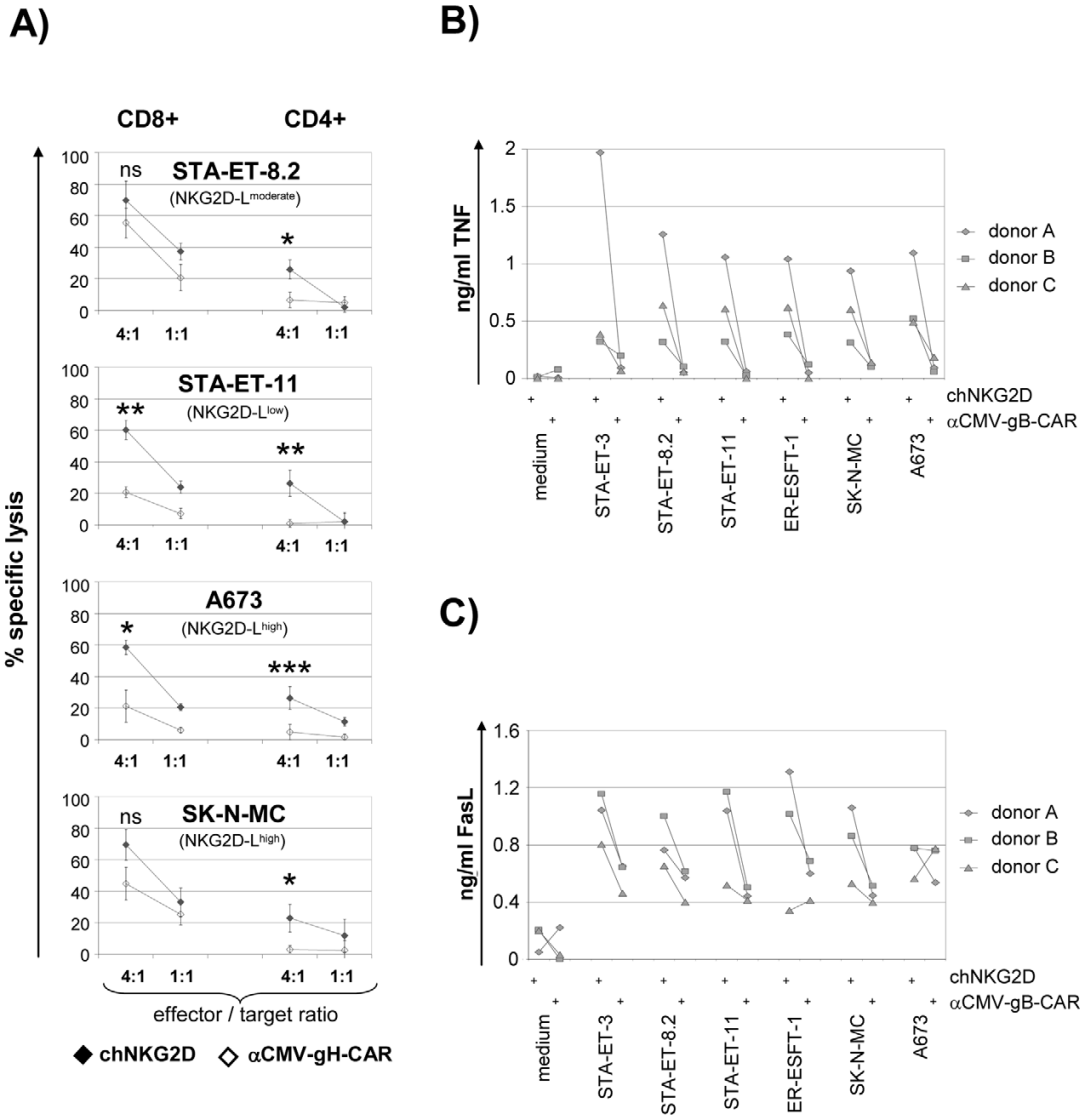
Targeting ESFT cells by chNKG2D-mRNA transfected T cells. *A,* Lysis of ESFT cells. The diagram shows the lytic activity of anti-CD3-activated CD8^pos^ and CD4^pos^ T cells transfected with mRNA encoding either chNKG2D or a similar chimeric CMV-gH-specific control receptor (αCMV-gH-CAR) in response to ESFT cells with different ligand expression (stated in brackets). Displayed is the specific lysis of the indicated ESFT cell lines at the indicated E∶T ratio (measured by Eu-release; mean ± S.D. of triplicates). Statistical significance was calculated for the effects of T cells expressing the chNKG2D versus T cells expressing the control receptor where indicated. *B and C,* Cytokine secretion of chNKG2D-mRNA transfected CD4^pos^ T cells in response to ESFT cells. The diagrams show TNF and FasL secretion by CD4^pos^ T cells transfected with RNA encoding chNKG2D or a CMV-gB-specific control receptor in response to the indicated cell lines or only medium as control (measured by ELISA; mean of duplicates).

In the following experiments we tested the capacity of RNA transfected activated T cells to recognize ESFT cells with varying and partially low ligand density (Fig. 1). Figure 3A illustrates the lysis of ESFT cells by T cells expressing either chNGK2D or an irrelevant CMV-specific control receptor. ESFT cells were specifically lysed by chNKG2D^pos^ T cells. Notably, chNKG2D^pos^ CD4^pos^ T cells lysed over 20% of ESFT cells at an E∶T ratio of only 4∶1 and even targets with low-level ligand density such as STA-ET-8.2 and -11. Figures 3B and 3C demonstrate that chNKG2D also mediated specific cytokine release in mRNA transfected CD4^pos^ T cells, as shown by detection of TNF and soluble FasL. Together, these results confirm that chNKG2D-RNA transfected T cells specifically recognize ESFT cells even with low ligand density. Furthermore, the data suggest that also the CD4^pos^ subset of T cells could mediate an efficient anti-ESFT response.

### Lentiviral expression of chNKG2D

**Figure 4:**
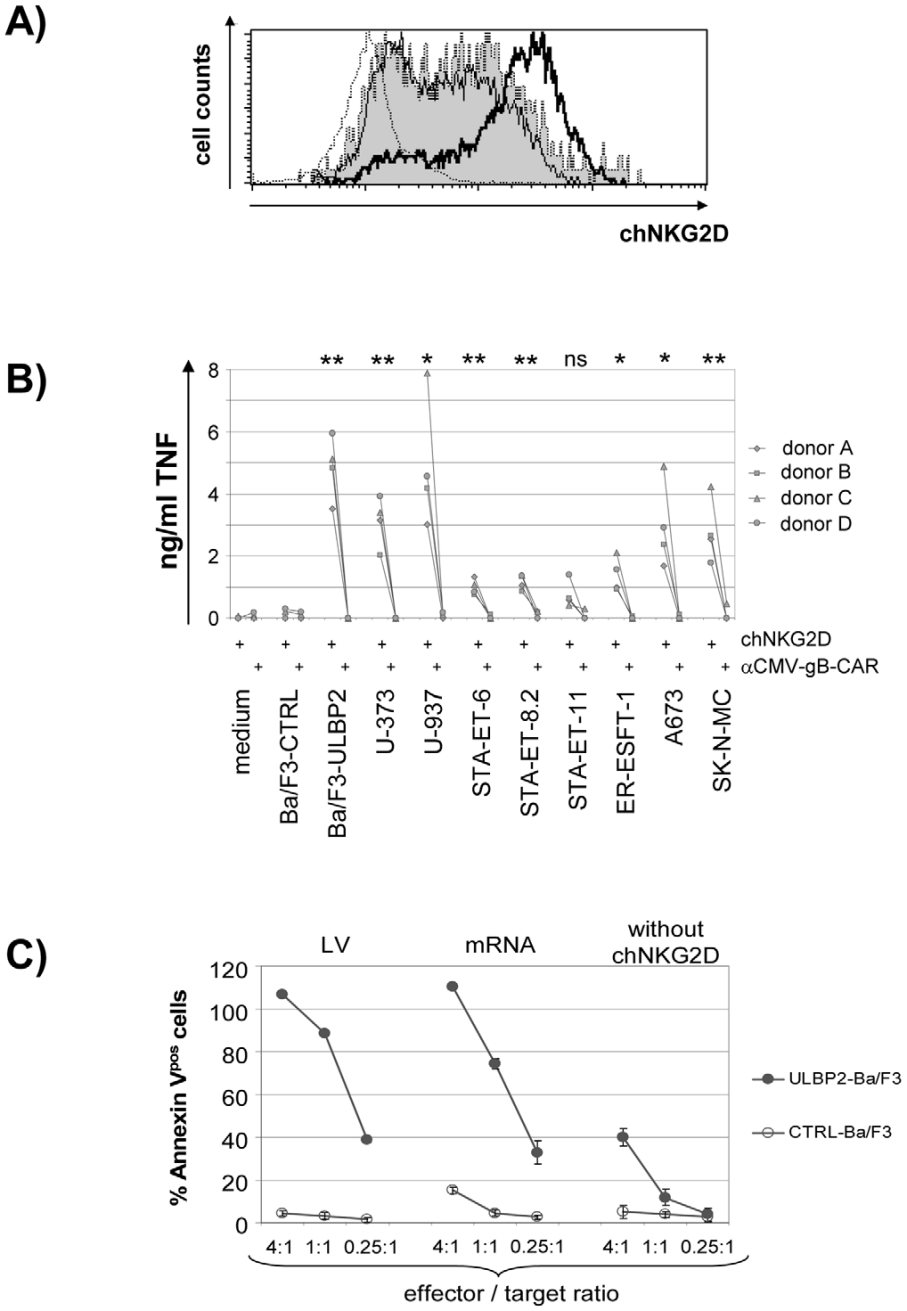
Lentiviral transduction for transfer of chNKG2D. *A,* antigen-specific enrichment of lentivirally transduced chNKG2D^pos^ MNCs. On day 13 after transduction the cells were co-cultured either with Ba/F3, ULBP2^pos^ Ba/F3 or without target cells. FACS analysis was performed 6 days later (open histogram, bold line: co-culture with the ULBP2^pos^ Ba/F3, 86.7% chNKG2D^pos^ T cells; filled histogram, dotted line: co-culture with Ba/F3, 60.7% chNKG2D^pos^ T cells; thin line: cells cultured without target cells, 54.1% chNKG2D^pos^ T cells; open histogram, dotted line = secondary antibody only). *B,* cytokine production. The diagram shows TNF secretion of purified CD4^pos^ T cells lentivirally transduced with either chNKG2D (enriched to 83–92% positive cells) or a similar chimeric CMV-specific control receptor (enriched to 69–81%) in response to the indicated cell lines or only medium as control (measured by ELISA; mean of duplicates). Ba/F3 or ULBP2^pos^ Ba/F3 cells were used for negative and positive control. Statistical significance was calculated for the effects of T cells expressing the chNKG2D versus T cells expressing the control receptor. *C,* Comparison of the cytotoxic response triggered by chNKG2D either stably or transiently transferred. CD8^pos^ T cells either unmodified (“without chNKG2D”) or expressing chNKG2D by lentiviral transduction (“LV”; enriched for chNKG2D^pos^ >90%) or mRNA transfection (“mRNA”; 20 hours after electroporation) were co-cultured with dye-labeled murine Ba/F3 cells expressing human ULBP2 (ULBP2-Ba/F3) or not (CTRL-Ba/F3). The diagram shows the percentage of Annexin V^pos^ Ba/F3 target cells after 4 hours of co-culture at the indicated E∶T ratios (mean ± S.D. of triplicates).

For lentiviral expression we cloned chNKG2D into the bicistronic vector pWPI, which was previously optimized for high-level transgene expression in hematopoietic cells [29]. Transduction of PHA-activated T cells yielded expression of chNKG2D in different cell types at similar proportions (16.1% of chNKG2D^pos^ CD8^neg^ cells and 19.3% of chNKG2D^pos^ CD8^pos^ cells in a representative experiment, data not shown). After removal of PHA but in the continued presence of IL-2 chNKG2D^pos^ cells enriched during prolonged culture (data not shown), suggesting an auto-stimulatory effect possibly through endogenous expression of NKG2D-Ls induced by PHA stimulation [30]. Accelerated enrichment and expansion was achieved by restimulation with an ULBP2^pos^ murine Ba/F3 cell line (Fig. 4A). Repeated restimulation in IL-2 supplemented medium resulted in highly enriched (>80%) chNKG2D^pos^ CD8^pos^ and CD4^pos^ T cells that could be expanded for at least three months. Expression of the receptor was stable during the whole period of restimulation, and waned only gradually over a time period of few weeks after cessation of periodical restimulation (data not shown). Figure 4B shows the specific TNF production of enriched chNKG2D^pos^ CD4^pos^ T cells in response to different NKG2D-L^pos^ tumor cells. Finally, we directly compared the lytic activity of CD8^pos^ T cells modified with chNKG2D either by lentiviral gene transfer or by mRNA electroporation. As shown in Figure 4C, both transfer methods conferred lytic capacity to a comparable extent against ULBP2 expressing target cells but not against ligand negative control cells. Not surprisingly, we observed also weak lysis of ULBP2^pos^ target cells, but not of ULBP2^neg^ control cells, when co-cultured with receptor unmodified CD8^pos^ effector cells, which we ascribe to endogenous expression of (costimulatory) CD314 (NKG2D).

Together the data demonstrate that lentiviral transduction allowed ligand induced long-term proliferation and the generation of highly enriched chNKG2D^pos^ T cells, which were characterized by a strong NKG2D-L specific responsiveness comparable to that observed with mRNA modified T cells.

### Stability of chNKG2D surface expression upon target cell recognition

**Figure 5:**
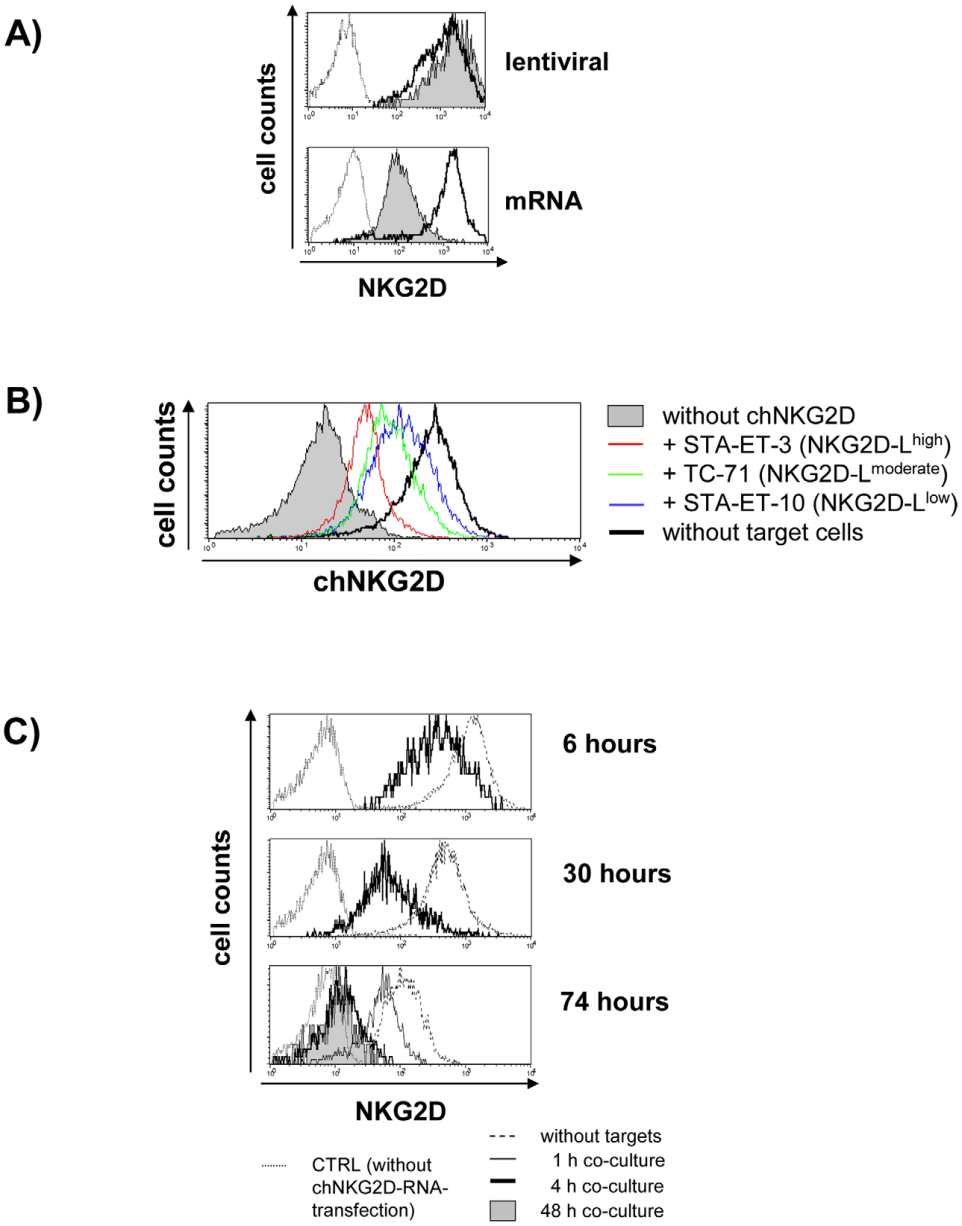
Stability of receptor surface expression upon target-cell encounter. The histograms show the flow cytometric analysis of chNKG2D expression (detected in CD4^pos^ T cells by an anti-CD314 antibody or in CD8^pos^ T cells by an anti-human-IgG1-Fc antibody; log scale in all histograms; note: amplification voltage in flow cytometric analysis was lowered for lentivirally transduced T cells due to the strong expression of the receptor). *A,* comparison of lentivirally transduced and mRNA transfected T cells. 75,000 chNKG2D^pos^ CD4^pos^ T cells were cultured 24 hours after electroporation for further 24 hours in presence or absence of 200,000 STA-ET-3 cells (filled grey histograms = presence of STA-ET-3, open histograms bold line = absence of STA-ET-3, open histograms dotted line = T cells not transfected). Shown is one representative experiment out of three independent repetitions. *B,* chNKG2D down-regulation in response to different ESFT cell lines. 100,000 chNKG2D^pos^ CD8^pos^ T cells were co-cultured 50 hours after mRNA electroporation for further 24 hours with 100,000 cells of different ESFT cell lines as indicated (unmodified T cells = “without chNKG2D”; without co-culture = “without target cells”). *C,* kinetics of chNKG2D down-regulation upon target-cell encounter at different times after transfection. 20,000 chNKG2D^pos^ CD4^pos^ T cells were co-cultured with 60,000 STA-ET-3 target cells for different times (1, 4, or 48 hours) before chNKG2D expression was determined (6, 30, or 74 hours after transfection) (one experiment). *D,* comparison of differentially stabilized mRNA species. ChNKG2D^pos^ CD4^pos^ T cells were co-cultured with STA-ET-3 target cells at different effector∶target ratios and at different times after transfection. Target cells were added at the indicated ratios either two or 26 hours after transfection and chNKG2D expression was determined by flow cytometry 6, 30, 50, or 74 hours after transfection. Shown is one out of three experiments. *E,* effect of patient serum containing soluble NKG2D-Ls. 15,000 chNKG2D^pos^ CD4^pos^ T cells were cultured 24 hours after electroporation for further 24 hours with or without serum of two different neuroblastoma patients (final serum concentration 33%).

Our data so far confirmed that both mRNA transfection and lentiviral transduction mediated high level expression of the chNKG2D in T cells. However, ligand-induced receptor internalization, which is known for T cell receptors (TCRs) and has been reported also for CARs, could result in a significant loss of receptor molecules from the cell surface upon target cell recognition [31]. For this purpose, we compared the stability of chNKG2D surface expression obtained by either RNA electroporation or by lentiviral transduction, after co-culture with MICA^pos^/ULBP2^pos^ STA-ET-3 target cells (Fig. 5A). Co-culture of the RNA-modified cells one day after electroporation with a 2.7 fold excess of STA-ET-3 target cells for 24 hours led to a reduction of the surface expression of chNKG2D by more than 90% compared to the expression observed in T cells without co-culture, as estimated by peak channel numbers (without co-culture: 1762; with co-culture: 87). This was in contrast to lentivirally modified T cells, in which chNKG2D expression even slightly increased during co-culture. As the extent of CAR internalization is strongly determined by the target antigen density [31], [32], we investigated the effect of interaction with ESFT cell lines characterized by different levels of NKG2D-L expression. Figure 5B illustrates that chNKG2D down-modulation in mRNA transfected cells indeed correlated with ligand density on ESFT cells.

In the following experiments we investigated the influence of the ESFT cell line STA-ET-3, most effective in CAR down-modulation with mRNA transfected T cells, in more detail. Figure 5C illustrates, that the capacity of mRNA-electroporated T cells to compensate for ligation induced chNKG2D down-regulation strongly decreased already early after transfection. Target-cell encounter three days after transfection almost completely down-modulated chNKG2D from the cell surface within only 4 hours of contact at an E∶T ratio of 1∶3. Finally, we compared two differentially engineered RNA species encoding chNKG2D for their capacity to compensate receptor down-modulation by lower number but prolonged presence of target cells (Fig. 5D). RNA #1 refers to the RNA used in all experiments described above and RNA #2 was modified according to Holtkamp et al. with RNA stabilizing elements [25]. The figure illustrates the comparably slow decline of chNKG2D expression over a time period of 6, 30, 50, and 74 hours after mRNA transfection, when T cells were cultured without target cells (open histograms, bold line). A markedly accelerated decline occurred, when target cells were added 2 or 26 hours after transfection. The receptor was strongly down-regulated at time point 30 hours after transfection, when tumor cells were added 2 hours after transfection at an E∶T ratio of 1∶1 (filled histograms). Addition of tumor cells at later time points (26 hours after transfection) resulted in more pronounced chNKG2D down-regulation at time point 50 hours after transfection. Then, even lower amounts of tumor cells (E∶T 1∶0.33; open histograms, thin line) resulted in marked down-regulation, which after an additional day of co-culture (FACS analysis 74 hours after transfection) was very similar to cultures with an E∶T of 1∶1. Importantly, almost identical effects were observed with both types of mRNA, although RNA #2 slightly mitigated the ligation induced down-modulation (visible at E∶T 1∶0.33). Together, these experiments had shown that the extent of down-modulation correlates with target cell numbers and increases with time following transfection.

It has been reported that endogenously expressed NKG2D is down-regulated by cell-bound ligands but not by soluble ligands [33], [34]. In view of the observed reduction of chNKG2D cell surface density in mRNA-transfected T cells, we wanted to investigate this issue with sera from tumor patients, which are known to frequently contain high levels of a soluble fraction of different NKG2D-Ls and thereby could inactivate injected T cells. Thus, we co-cultured RNA-modified CD4^pos^ T cells with two sera of neuroblastoma patients containing high detectable levels of MICA (171 and 494 pg/ml; “NB1” and “NB2” respectively). In these experiments, however, we did not observe any reduction of chNKG2D surface density (Fig. 5E).

## Discussion

The goal of our study was to evaluate the potential of a T cell based approach for adoptive therapy of EFST using a chimeric NKG2D receptor and to compare the suitability of two different methods for receptor transfer. For this aim we investigated NKG2D-Ls, which have previously been reported to be expressed in ESFT [4], as potential ESFT target antigens. In confirmation and extension of Verhoeven et al.'s data, we found NKG2D-Ls consistently expressed in all of 14 tested ESFT cell lines (Fig. 1). Based on this and on the fact that our chNKG2D modified T cells efficiently recognized ESFT cells also with low NKG2D-L expression, we propose a chNKG2D based approach for immunotherapy of ESFT lesions.

Originally, the feasibility of redirecting T cells to NKG2D-Ls has been demonstrated by Sentman et al. using NKG2D fused as a full-length protein to the cytoplasmic domain of CD3ζ [11]–[14]. For our study, we intended to construct a NKG2D-based CAR with an integrated costimulatory CD28 domain for enhanced T cell activation [8], [15]. Notably, CD28 signaling enhances the resistance to TGF-β1 and regulatory T cells (Tregs) and thus could be particularly relevant in metastasized ESFT, for which secretion of TGF-β1 and increased numbers of infiltrating Tregs have been described [20], [35], [36]. Further, signal amplification by CD28 reduces the threshold of endocytosed receptor molecules required for T cell activation [37], and, hence may be favorable for targeting ESFT, which express NKG2D-Ls often at rather low levels (Fig. 1). Low target antigen density may impair the activation of CAR modified T cells, as CARs require about 100 fold higher target antigen densities than T cell receptors to activate a T cell, which is explained by the lack of serial triggering due to high affinity interaction [31], [32], [37], [38]. Specifically, using a first generation CAR, James et al. have shown that it requires ∼30,000 target molecules/target cell (inducing endocytosis of ∼20,000 CAR molecules) to trigger maximum lytic activity [31], [32]. Increased ligand densities may be required if receptor expression is low [39].

In line with these theoretical considerations we indeed found that CD8^pos^ T cells redirected by our novel type of chNKG2D at high expression levels could efficiently lyse ESFT cells even when their ligand expression was low (e.g. STA-ET-11; Fig. 3A). This might be important and appears to be in line with the reported NK cell lysis of ESFT cells, for which also no relation between susceptibility to lysis and the expression levels of the triggering ligands has been reported [5].

Activated NK cells have been shown to kill ESFT cells with high efficiency [4], [5]. By using a flow cytometry-based method, Cho et al. have observed up to 87% propidium iodide positive cells within 4 hours of co-culture at an E∶T ratio of 1∶1. In our study we relied on a cytotoxicity-assay based on the release of non-radioactive Europium (Fig. 3A) or flow-cytometry using Annexin V staining (Figs. 2C and 4C). In general, the Annexin V based method seems to give higher estimates of cytotoxicity (data not shown), probably due to the fact that Annexin staining detects early steps of apoptosis. Taken into account the different methods used by this group and by us, our results with CAR modified T cells appear to be within the same magnitude of killing efficiency compared to the NK cells tested by Cho et al.

The cytotoxicity results shown in Figures 2C and 3A underline that CD4^pos^ T cells in addition to the CD8^pos^ subset are attractive candidates for expression of the chNKG2D. The observed strong cytotoxicity of CD4^pos^ T cells might be explained by several potentially cytotoxic proteins. We found that chNKG2D modified CD4^pos^ T cells in response to ESFT cells did not express detectable amounts of TRAIL or perforin (data not shown). However, we found soluble FasL in the supernatants of these cells (Fig. 3C), which may contribute to killing since ESFT cells are sensitive to Fas cross-linking (data not shown and [40]–[42]).

Apart from chNKG2D-dependent cytotoxicity, we repeatedly observed some chNKG2D-independent background killing by particularly CD8^pos^ T cells (unmodified or expressing an irrelevant control-CAR; Figs. 2C, 3A, and 4C) which we could not attribute to eventually contaminating NK cells (purity of the cell population after cell sorting and cell expansion: <1% NK cells; data not shown). Such CAR-independent cytotoxicity, however, may likely be due to endogenously expressed NKG2D and other activating receptors, particularly DNAM-1 (CD226), which has been described to mediate strong TCR-independent lysis of many tumor cell lines [43], [44]. Its ligands, CD155 and CD112, are also frequently up-regulated in tumors including ESFT. Together with NKG2D-Ls the ligands of DNAM-1 efficiently trigger NK cell cytotoxicity against ESFT cells [4], [21], [45] and likely also mediate some CAR-independent killing of ESFT by activated CD8^pos^ and CD4^pos^ T cells. With regard to the background FasL production observed also in cultures containing control-CAR transfected T cells (Fig. 3C), we cannot exclude a contribution from the ESFT cells themselves [46]. However, when cultured without T cells, our ESFT cell lines produced less than 60 pg/ml sFasL (data not shown).

For redirecting T cells we used RNA transfer and lentiviral transduction which both allowed high level expression of chNKG2D and comparable elimination of target cells. Regarding the persistence of the expression, it is known that transfected mRNA maintains substantial translation only within the first day after electroporation [25], but that the translated protein persists at high density on the cell surface for several days. According to our data, the persistence on the cell surface actually might be much more limited due to rapid ligand induced receptor internalization upon target cell recognition. Ligand induced receptor internalization is important for proper signal transduction [47], [48], and has been quantified for the T cell receptor complex and also for CARs [31], [37]. CARs are internalized by ligands roughly at a ratio of 1∶1 whereas 100 fold more TCR molecules per target antigen are internalized due to serial triggering.

Cross-linking of transmembrane receptors generally results in rapid redistribution from the cell surface into intracellular compartments, where a fraction of the receptors enters lysosomal degradation instead of being recycled back to the cell surface [47], [48]. As a consequence, a high density of ligand molecules on target cells can rapidly reduce the cell surface density of retrovirally expressed CAR molecules below the critical threshold required for T cell activation resulting in the loss of serial killing capacity [31].

Here we show that the strong lentiviral expression of our chNKG2D could effectively compensate ligand induced internalization in response to STA-ET-3 cells, expressing several different NKG2D-Ls (Figs. 1 and 5A), whereas mRNA transfection was similarly effective only during the first hours after electroporation (Fig. 5D). Target cell contact at later time points, however, resulted in strong and irreversible down-modulation of chNKG2D (Figs. 5C and 5D). Notably, this was seen with mRNA, for which we had applied state of the art techniques to maximize stability and translation, i.e. extensive polyadenylation and capping with a specially modified cap analog (see materials and methods) [25], [49]. Further attempts to improve mRNA stability according to Holtkamp et al. yielded only negligible effects (“RNA #2” in Fig. 5D) [25], [49].

Such characteristics of mRNA mediated CAR expression could be significant, since clinical studies have shown, that intravenously administered T cells in humans due to extensive organ trapping and long migration distances reach tumor sites only two or three days after infusion [50], [51]. Our observations would argue for injecting mRNA transfected T cells in humans not only repetitively but also perhaps intra-tumorally or intra-arterially to ensure that the T cells meet their targets without any further delay.

Regardless of the applied receptor transfer strategy, our data illustrate for the first time the potential of an NKG2D based approach for targeting ESFT cells by receptor modified T cells. The new receptor efficiently triggered the elimination of ESFT cells *in vitro* by CD8^pos^ and CD4^pos^ T cells and thereby opens a new avenue for developing an adoptive T cell approach that could have the potential to complement conventional multimodal therapy of ESFT.
